# Flavonoids as Cytokine Modulators: A Possible Therapy for Inflammation-Related Diseases

**DOI:** 10.3390/ijms17060921

**Published:** 2016-06-09

**Authors:** Nayely Leyva-López, Erick P. Gutierrez-Grijalva, Dulce L. Ambriz-Perez, J. Basilio Heredia

**Affiliations:** 1 Centro de Investigación en Alimentación y Desarrollo A.C., Carretera a Eldorado Km 5.5 Col. El Diez, 80110 Culiacán, Sinaloa, Mexico; nayely061005@gmail.com (N.L.-L.); erickpaulggrijalva@gmail.com (E.P.G.-G.); 2 Universidad Politécnica del Mar y la Sierra, Carretera a Potrerillos del Norote/La Cruz Km 3, La Cruz, 82740 Elota, Sinaloa, Mexico; dulceambriz@hotmail.com

**Keywords:** flavonoids, anti-inflammatory effect, cytokine inhibition

## Abstract

High levels of cytokines, such as interleukin (IL)-1β, tumor necrosis factor (TNF)-α and IL-6, are associated with chronic diseases like rheumatoid arthritis, asthma, atherosclerosis, Alzheimer’s disease and cancer; therefore cytokine inhibition might be an important target for the treatment of these diseases. Most drugs used to alleviate some inflammation-related symptoms act by inhibiting cyclooxygenases activity or by blocking cytokine receptors. Nevertheless, these drugs have secondary effects when used on a long-term basis. It has been mentioned that flavonoids, namely quercetin, apigenin and luteolin, reduce cytokine expression and secretion. In this regard, flavonoids may have therapeutical potential in the treatment of inflammation-related diseases as cytokine modulators. This review is focused on current research about the effect of flavonoids on cytokine modulation and the description of the way these compounds exert their effect.

## 1. Introduction

Flavonoids are natural occurring compounds with a wide range of molecular diversity, and more than 10,000 structures have been reported [1]. Fruits, vegetables, herbs and other plant food are all flavonoid sources [2]. The interest in flavonoids has arisen because the intake of these compounds has been associated with the prevention and treatment of diseases, which is translated to benefits in health [2,3,4]. The anti-inflammatory effect of flavonoids is one important biological activity.

The activity of flavonoids in the inflammatory response include the inhibition of inflammatory mediators like reactive oxygen species (ROS) and nitric oxide (NO); the regulation of activity of inflammatory enzymes, such as cyclooxygenases (COXs) and inducible nitric oxide synthase (iNOS); the reduction in levels of production and expression of cytokines and the modulation of transcription factors, such as the nuclear factor κ-light-chain-enhancer of activated B cells (NF-κB) and activating protein-1 (AP-1) [5,6,7,8]. When the inflammatory response is not regulated, it results in an increase in the concentration of inflammatory mediators, which might lead to the occurrence of several chronic diseases, namely rheumatoid arthritis, coronary diseases and cancer, among others [9,10]. There is evidence to suggest that inflammatory cytokines have potential as therapeutic targets to treat inflammatory diseases [11], therefore, studying the effect of flavonoids on inflammatory mediators, especially by modulating cytokines, is relevant in order to develop alternative treatments for inflammation-related diseases.

The present manuscript will review recent evidence regarding the role of flavonoids as modulators of inflammatory mediators, mainly cytokines, associated with the inflammatory response.

## 2. Inflammatory Response

Inflammation is a response of the organism to the presence of diverse agents, such as pathogens (bacteria, fungi and viruses), trauma (shock or burns), toxic compounds (pollutants), as well as reactions of the immune system (hypersensitivity) in the body, which causes a disruption of tissue homeostasis [12,13]. There are two types of inflammation, acute or chronic. The former is the initial response to injury and lasts a few hours or a few days. When acute inflammation does not successfully eliminate damaging agents it can cause a chronic phase. Chronic inflammation is an extended inflammation period (weeks to months) and is associated with the presence of lymphocytes and macrophages, vascular proliferation, fibrosis and tissue destruction. Moreover, chronic inflammation has been related to diseases, such as rheumatoid arthritis, asthma, atherosclerosis, Alzheimer’s disease and cancer, among others [14,15,16].

During inflammation, cells of the immune system, mainly macrophages, could be activated through the recognition of a pathogen endotoxin, lipopolysaccharide (LPS), by Toll-like receptors (TLR). This event provokes a signaling pathway that will release the NF-κB, that activates genes associated with the transcription of proteins related to the inflammatory process, such as iNOS, responsible for NO synthesis, COXs, which synthetize prostaglandins, and cytokines. The TLR signaling pathway also triggers the generation of ROS [14,17,18,19]. Activating protein-1 (AP-1) is another transcription factor that is important during the inflammatory response. This factor responds to a wide variety of stimuli such as bacterial and virus infection, stress and growth factors leading to the regulation of gene expression of pro-inflammatory mediators, including cytokines [20]. Overproduction of some inflammatory mediators, such as cytokines, as a result of chronic inflammation, might lead to the occurrence of several chronic diseases [21,22,23].

## 3. Cytokines

Cytokines are proteins that play an important role in the inflammatory response. The majority of the cytokines are produced by activated lymphocytes and macrophages, although endothelial and epithelial cells are able to produce these proteins, too. Cytokine expression is regulated by NF-κB and AP-1 and may be triggered by LPS, ROS and microbial species, among others [24]. Interleukin (IL)-1β and tumor necrosis factor (TNF)-α are two of the main cytokines involved in the inflammatory response. Briefly, these cytokines induce the expression of adherence molecules in endothelial tissue; participate in the synthesis of other cytokines, namely IL-6, and chemokines (IL-8 and monocyte chemoattractant protein-1 (MCP-1)), growth factors, eicosanoids and NO [14]. Other relevant cytokines are IL-10 (anti-inflammatory cytokine), IL-4 and IL-3, which together downregulate pro-inflammatory signals [6,25].

Cytokines can be classified into two groups: those related to acute inflammation and those responsible for chronic inflammation [26]. The cytokines involved in acute inflammation are IL-1, TNF, IL-6, IL-11, IL-8, IL-16 and IL-17, among others, whereas cytokines related to chronic inflammation are those mediating humoral responses, like IL-4, IL-5, IL-6, IL-7 and IL-13; additionally, there are cellular responses, namely IL-1, IL-2, IL-3, IL-4, IL-7, IL-9, IL-10 and IL-12, interferons (IFN), transforming growth factor (TGF)-β and TNF-α [26].

It has been proposed that a prolonged overproduction of inflammatory cytokines without regulation might lead to incidence of chronic diseases, such as, rheumatoid arthritis, atherosclerosis and Alzheimer’s disease, among others [15,16,27]. So the study of these proteins as biomarkers in inflammation-related diseases is of relevance to determine adequate treatment.

### Cytokines as Biomarkes in Inflammation-Related Diseases

Of all the cytokines associated to chronic inflammation that were mentioned before, this review will focus on IL-1β, TNF-α and IL-6, due to the fact that these are the most well-studied and have a predominant role in chronic inflammation-related diseases. Among the chronic inflammation-related diseases that have shown to be associated to these cytokines are: rheumatoid arthritis; atherosclerosis; metabolic syndrome and associated type 2 diabetes; neurodegenerative disorders such as Alzheimer’s disease, and; some forms of cancer in which inflammatory reactions promote tumor development [28]. Experimental research has linked cytokines IL-1β, TNF-α and IL-6 with several chronic inflammation-related diseases. For example, in Alzheimer’s disease, the inflammatory response in neurons includes activation of microglia (cells that protect neuronal function), astrocytes, macrophages and lymphocytes, resulting in the release of cytokines and other inflammatory mediators [29,30]. The release of these inflammatory mediators leads to the further release of more inflammatory factors, as well as the recruitment of monocytes. In this sense, the inflammatory response contributes to the progress of Alzheimer’s disease accelerating the course of the disease. When microglia are activated, it results in an increased secretion of pro-inflammatory cytokines like IL-1β, IL-6 and TNF-α, thereby enhancing the ability of monocytes to pass through the blood-brain barrier [29,30].

IL-6, along with its receptor sIL-6Ralfa, commands the change from acute to chronic inflammation by shifting the nature of leucocyte infiltrate from polymorphonuclear neutrophils to monocyte/macrophages [31]. Because of the latter, IL-6 is known to be associated with chronic inflammation and related diseases. For example, elevated serum IL-6 levels have been detected in patients with systemic cancers, rheumatoid arthritis, systemic lupus erythematosus, psoriasis and Crohn’s disease as compared to healthy controls or patients with benign diseases [32,33,34,35,36]. It has been demonstrated that IL-6 is secreted by many types of cancer cells as it occurs in renal cell carcinoma. IL-6 is abundant in the serum of 50% of the patients with metastatic renal cell cancer; moreover, these cancer cells have shown the production of IL-6 and expression of IL-6 mRNA and of the soluble and membrane-bound gp120 chain of the IL-6 receptor [37,38]. This has turned IL-6 into a drug target in the treatment of chronic inflammatory diseases [26,31], since the inhibition of IL-6 and its signaling cascade was effective as treatment regimen in studies of inflammatory diseases [31,34,39,40].

IL-1β, in conjunction with other inflammatory mediators, has shown to be induced by the activation of microglia cells, which can lead to neuronal death, and thus to the progression of Alzheimer’s disease [29,30,41]. Additionally, higher levels of serum IL-1β have been found in patients with abdominal obesity and periodontitis [42]. Another example of the role of cytokines in chronic diseases can be found between TNF-α and rheumatoid arthritis, where anti-TNF-α antibodies were added to *in vitro* cultures of cells from diseased joints and inhibited the production of IL-1β and other cytokines. Additionally, the use of TNF-α inhibitors has demonstrated remarkable efficacy in the control of diseases’ signs and symptoms [43]. Moreover, in Alzheimer’s disease, during amyloid beta-peptide aggregation, microglia cells are activated and thus the production of TNF-α is stimulated, promoting neuronal death [29,41,44]. IL-1β and TNF-α are produced by activated macrophages, as well as mast cells, endothelial cells, and some other cell types. The principal role of these cytokines in inflammation is in endothelial activation. Both IL-1β and TNF-α stimulate the expression of adhesion molecules on endothelial cells. This increases leukocyte binding and recruitment, and enhance the production of additional cytokines and eicosanoids. TNF-α also increases tissue fibroblasts, resulting in increased proliferation and production of extracellular matrix [14,43,45].

Because of the important role of cytokines, and other inflammatory mediators, in the development of diseases like rheumatoid arthritis and cancer, there have been efforts looking for pharmaceutical drugs to treat inflammation-related diseases.

## 4. Anti-Inflammatory Drugs

There are two main types of anti-inflammatory drugs: the nonsteroidal anti-inflammatory drugs (NSAIDs), which inhibit COX activity, and cytokine receptor inhibitors, which block cytokine activity. Examples and the mode of action of these anti-inflammatory drugs will be mentioned next.

### 4.1. Nonsteroidal Anti-Inflammatory Drugs (NSAIDs)

Nonsteroidal anti-inflammatory drugs (NSAIDs) are widely prescribed and come in different chemical groupings [46,47]. It has been reported that all the NSAIDs drugs act by inhibiting COX enzymes, which are involved in inflammation and are responsible for the synthesis of prostaglandins involved in normal physiological processes. The inhibition of these actions is responsible for the majority of the adverse effects of NSAIDs in clinical use, and for their main toxicity and overdose [46,48]. All NSAIDs have been reported to increase the risk of gastrointestinal damage; the most common side effects range from benign dyspepsia and esophagitis to upper-gastrointestinal bleeding, perforation, and gastric outlet obstruction [49,50,51].

### 4.2. Cytokine Receptor Inhibitors

The cytokine receptor inhibitors are drugs based on the premise that, in order to function, cytokines must bind to specific receptors. Some cytokines have one receptor chain, like type I interferons, whilst other cytokines bind to shared receptors, like IL-4 and IL-13. In this sense, the mechanism of action of cytokine receptors is not yet well understood, although it is thought that receptors are pre-assembled on the cell surface and are activated by structural changes in the receptors upon binding [52,53,54,55].

On this subject, several drugs have been developed to inhibit cytokine activity. These include the inhibitors of TNF-α and IL-1β with different modes of action [55]. For example, Etanercept, Infliximab and Anakinra are drugs that bind to TNF-α and IL-1 receptors, respectively [52,55].

Moreover, in the treatment of rheumatoid arthritis, several drugs have been used; among the most common are the biologic disease-modifying antirheumatic drugs (bDMARD) or TNF-α inhibitors. However, even with these drugs, around 20%–40% of patients have shown an inadequate response. An alternative is the use of Tocilizumab, a humanized anti-IL-6R monoclonal antibody that prevents IL-6 from binding to its receptor IL-6R [56,57,58,59]. Some other drugs have been studied with the purpose of blocking cytokine actions, and some of these are summarized in Table 1 [60].

Due to its importance in the progression of chronic inflammatory diseases, the control of cytokine action is still a major focus of drug and pharmaceutical research. With efforts in developing cytokine antagonists like cytokine receptor blockers, it is worthwhile to mention that cytokine receptor inhibitors have secondary effects. For example, when Tocilizumab, an anti-IL-6 receptor widely used in the treatment of rheumatoid arthritis, is used in combination with disease-modifying antirheumatic drugs, an elevation in cholesterol and alanine aminotransferase levels have been reported [61]. On the other hand, Anakinra has not shown any adverse effects when used in patients with acute gouty arthritis, while some other therapeutic agents such as Ustekinumab, Etanercept and daclizumab have proven not to be effective against multiple sclerosis [62].

Due to secondary effects that occur when using anti-inflammatory drugs on a long-term basis, it is primordial to find alternative therapies to treat inflammatory diseases. Natural compounds, such as flavonoids, are among the studied molecules in alternative research treatment for inflammation-related illness.

## 5. Flavonoids and Their Anti-Inflammatory Properties

**Figure 1:**
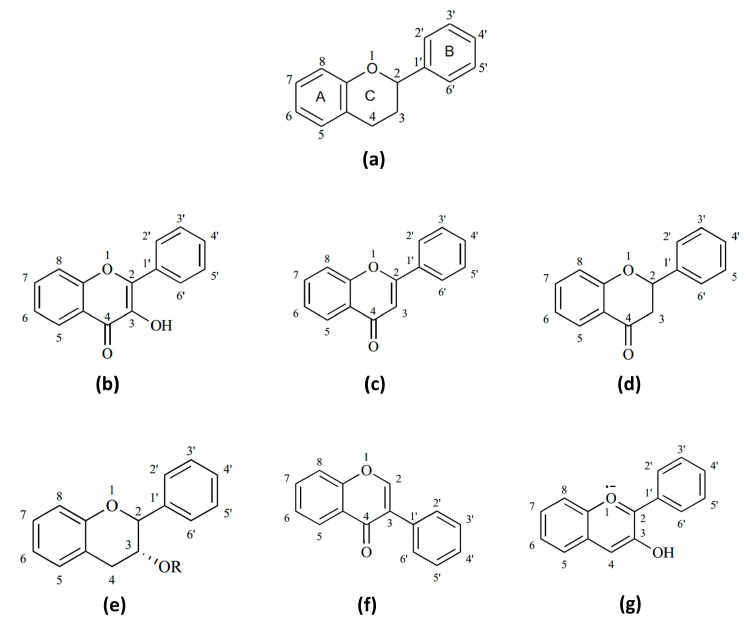
Basic chemical structure of different flavonoid classes. Structure of (**a**) basic flavonoid skeleton; (**b**) flavonols; (**c**) flavones; (**d**) flavanones; (**e**) flavanols; (**f**) isoflavones and (**g**) anthocyanidins.

Flavonoids are natural compounds with a common C6–C3–C6 structure containing two aromatic rings linked by a three carbon chain, typically organized as an oxygenated heterocyclic ring (Figure 1) [70]. The main classes of flavonoids are flavonols, flavones, flavanones, flavanols, isoflavones and anthocyanidins [71]. These compounds are produced as secondary metabolites by plants as defense mechanism against biotic and abiotic stress conditions, mainly [70]. Furthermore, it has been extensively demonstrated that flavonoids possess a wide range of health benefits due to their nutraceutical properties such as antibacterial, antioxidant and anti-inflammatory, among others [8,72,73]. The anti-inflammatory potential of flavonoids is of particular interest for the purpose of this review.

It has been well established that flavonoids have a similar mechanism of action to NSAIDs. In addition, flavonoids inhibit the activity or gene expression of other pro-inflammatory mediators aside from COX. Indeed, flavonoids can up/down regulate transcriptional factors in inflammatory and antioxidant pathways, like NF-κB and Nrf-2 [74].

**Figure 2:**
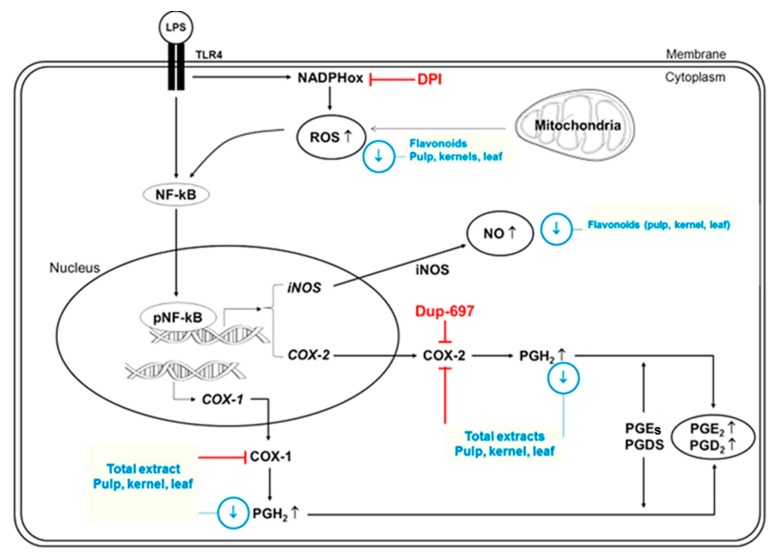
Hypothetical model that shows the possible effect of flavonoids from *J. platyphylla* on the levels of some inflammatory mediators. Lipopolysaccharide (LPS) binds to TLR4 receptor and triggers the generation of reactive oxygen species (ROS) from nicotinamide adenine dinucleotide phosphate (NADPH)-oxidase and mitochondria. ROS-mediated redox reactions activate the nuclear translocation of the nuclear factor κ-light-chain-enhancer of activated B cells (NF-κB). The NF-κB activation mediates inducible nitric oxide synthase (iNOS) and cyclooxygenase (COX) expression. Both COX-1 and COX-2 activities mediate the production of prostaglandins. In addition, *J. platyphylla* total extracts (mixture of flavonoids and lipophilic compounds) inhibited both COX-1 and COX-2 activities. The up black arrow indicates an increase on inflammatory mediators when macrophage cells are stimulated with LPS. The down blue arrow shows suppressive effect on ROS, NO and prostaglandin levels by flavonoids. The red T-shaped symbol indicates inhibition on protein activity. Adapted from Ambriz-Perez, Bang, Nair, Angulo-Escalante, Cisneros-Zevallos and Heredia [76].

In this regard, polyphenols presented anti-inflammatory activity in LPS-induced inflammation in RAW 264.7 macrophage cells. Flavonols from *C. ternatea* exhibited a strong suppression of COX-2 activity and partial ROS inhibition, while its ternatin anthocyanins inhibited nuclear NF-κB translocation, iNOS protein expression, and NO production [75]. Flavonoids, such as apigenin, genistein, and luteolin glycosides from *J. platyphylla*, an endemic plant from Mexico, showed potential as anti-inflammatory agents due to their significant inhibitory effects on ROS and NO levels produced by LPS-induced inflammation in RAW 264.7 mouse macrophage cells. The authors proposed a hypothetical mode by which flavonoids exert their anti-inflammatory role (Figure 2) [76]. Extracts from three Mexican oregano species, containing quercetin, luteolin and scutellarein glycosides, showed anti-inflammatory activity by lowering ROS and NO production in LPS-induced inflammation in RAW 264.7 macrophage cells [77]. Extracts from *Rhodomyrtus tomentosa*, containing the flavonoid quercetin, effectively suppressed the release of NO and prostaglandin E_2_ in LPS-treated RAW 264.7 cells and peritoneal macrophages [78]. These studies were about anti-inflammatory activity of plant extracts. Extracts are composed of a variety of flavonoids, so the bioactivity cannot be attributed to one specific flavonoid. Nevertheless, there are other studies in which the anti-inflammatory effect of individual flavonoids was evaluated.

In different mice models, apigenin (<10 μM) has shown inhibitory action on NO and prostaglandin E_2_ (PGE_2_) by inhibiting the expression of iNOS and COX-2, respectively. Furthermore, apigenin (25 mg/kg) suppressed p38 mitogen-activated protein kinase (MAPK) and c-Jun N-terminal kinase (JNK) phosphorylation without affecting the activity of extracellular signal-regulated kinase (ERK) [79]. Apigenin also played a protective role against hepatocarcinogenesis on lipid peroxidation, as an antioxidant defense [80]. Quercetin (10–25 μM) exerted an inhibitory effect on NO and TNF-α on BV-2 LPS-stimulated microglia cells [81]. Furthermore, quercetin (10 μM) down-regulated COX-2 and NF-κB expression and reduced NO production in ochratoxin-stimulated HepG2 (human hepatoma) cells [82]. Luteolin (<10 μM) inhibited NO, IL-6, MCP-1 and TNF-α production, as well as iNOS and COX-2 expression in pseudorabies virus-infected RAW 264.7 cells by inhibiting NF-κB activation [83].

The molecular mechanisms involved in the anti-inflammatory effect of flavonoids might include the inhibition of pro-inflammatory enzymes, such as COX-2 and iNOS; and cytokines, the inhibition of NF-κB, AP-1 and mitogen-activated protein kinase (MAPK) [84,85,86]. Evidence that supports this statement will be discussed next.

### Flavonoids as Anti-Cytokine Agents

A large number of phenolic compounds have been reported to inhibit both the secretion and expression of pro-inflammatory cytokines. Regarding the effect of flavonoids on cytokine secretion, it has been observed that apigenin, chrysin, diosmetin, kaempferol, luteolin, naringenin and quercetin, at 50 and 100 nM, reduced IL-6 and TNF-α secretion levels in LPS-stimulated RAW 264.7 macrophages [87]. The incubation of human periodontal ligament cells with apigenin (40 μM) significantly decreased the nicotine- and LPS-induced production of IL-1β, TNF-α, IL-6, and IL-12 [88]. Besides this, it was established that luteolin, quercetin, genistein, kaempferol, apigenin, diosmetin and hesperetin, at 25 and 50 μM, inhibited TNF-α release in LPS-activated macrophages, quercetin, luteolin and genistein being the most efficient inhibitors of this cytokine secretion. Furthermore, quercetin and luteolin exerted stimulatory effects on the expression of the anti-inflammatory cytokine IL-10, but at low concentrations (<50 μM) [89]. Luteolin (15–20 μM) has been proven to exert anti-cytokine effects on IL-1β and TNF-α release by murine BV-2 microgial cells stimulated with LPS/IFN-γ [90]. Moreover, luteolin (3–10 μM) significantly reduced IL-6 and TNF-α release by suppressing NF-κB activity in human monocytes under hyperglycemic conditions (20 mM glucose) [91].Naringenin (10–50 μM), extracted from *Nymphaea mexicana* Zucc, showed a noticeable inhibitory effect on NO, MCP-1 and TNF-α production in LPS-activated RAW 264.7 macrophages. Naringenin (10–25 μM) also inhibited LPS-mediated induction of protein expressions of iNOS, COX-2, and phospho-ERK [92]. Quercetin and Luteolin, both at 25 μM, effectively inhibited IL-1β, IL-6, IFN-γ and TNF-α production in human whole blood incubated with LPS [93]. A six-week supplementation of quercetin (150 mg) given to human subjects significantly decreased serum concentration of the cytokine TNF-α [94]. In relation to the effect of flavonoids on cytokine expression it has been established that quercetin (100–200 mg/kg), a very known anti-inflammatory flavonoid, reduced pancreatic histopathological damage and reduced the mRNA and protein expression of NF-κB, IL-1β, IL-6 and TNF-α in hypertriglyceridemia-related acute pancreatitis in rats [95]. Apigenin (20 mg/kg) administration to subarachnoid hemorrhage suffering rats significantly attenuated mRNA expression of TNF-α, IL-6 and IL-1β when compared to the untreated control, showing neuroprotective effects [96]. Fisetin (3–30 μM) reduced TNF-α, IL-1β, IL-6 and IL-8 expression and production in phorbol-12-myristate-13-acetate plus calcium ionophore (PMACI)-stimulated human mast cells. Additionally, fisetin inhibited phosphorylation of MAPKs and nuclear translocation of NF-κB induced by PMACI [97]. It has been demonstrated that luteolin-8-*C*-β-fucopyranoside (LU8C-FP) (50 μM) suppressed the expression levels of IL-6 on phorbol 12-myristate 13-acetate-treated THP-1 cells, a human leukemia monocytic cell line, by inhibiting MAPKs and NF-κB signaling pathways in human monocytic cells. Nevertheless, LU8C-FP failed to inhibit IL-1β and IL-8 expression, which provide information leading to the use of this flavonoid to treat inflammatory diseases caused by IL-6 [98], such as colitis, diabetes, rheumatoid arthritis, cancer and cardiovascular diseases [99,100,101,102,103].

NF-κB and AP-1 are important transcriptional factors in the modulation of pro-inflammatory mediators, like cytokines [85,104]. The first mediates the expression of cytokines and other inflammatory mediators [105], while the second participates in the synthesis of effector molecules and cytokines during innate immune response [106]. Due to the important role of NF-κB and AP-1 in inflammation, studies have been conducted in order to determine the effect of flavonoids in the modulation of these transcriptional factors. Quercetin (100 μM) significantly reduced high glucose-induced increased NF-κB and AP-1 activity by 43% and 69%, respectively, in rat aortic endothelial cells [107]. The treatment of IL-1β-induced human synovial sarcoma cells (SW982) with luteolin (1–10 μM) significantly reduced TNF-α and IL-6 production, inhibited JNK and p38 activation and diminished the activation of NF-κB and AP-1 transcription factors. These findings suggest that the flavonoid luteolin possess anti-cytokine activity in SW982 cells by inhibiting MAPKs (JNK and p38) and transcriptional factors (NF-κB and AP-1) [108].

As mentioned above, cytokine overproduction is highly related to chronic diseases such as Alzheimer’s disease, rheumatoid arthritis and cancer, among others [16,109,110]. Flavonoids being able to downregulate cytokine expression and secretion are a very promising alternative to be used as treatment of the diseases mentioned previously. A summary of the studies here addressed is shown in Table 2.

It has been proposed that anti-inflammatory mechanism of flavonoids is highly related to their chemical structure. The main features of flavonoids to exert their anti-inflammatory activity are: (I) a planar ring system in the flavonoid molecule; (II) unsaturation in the C ring at the C2–C3 position; (III) the number and position of hydroxyl groups at the A and B rings, particularly at C5 and C7 in A ring and at C3′ and C4′ in B ring; (IV) the lack of hydroxyl groups on B ring apparently eliminates the activity; (V) the keto group at C4 in C ring, and; (VI) non-glycosylation of the molecule [111,112]. In this regard, flavonoids with hydroxyl groups in 3′ and 4’ position, such as quercetin and luteolin, showed higher inhibitory effect on TNF-α release than those with only one hydroxyl group in B ring, namely genistein, regardless of the presence of a double bound in C2–C3 [89]. Luteolin exerts anti-inflammatory activity by inhibiting iNOS, IL-1β, IL-6 and TNF-α expression in LPS-stimulated RAW 264.7 macrophages, while *O*-glycosylated luteolin showed lower effect on iNOS and IL-1β expression than the aglycone [113]. To a better understanding of structure/anti-inflammatory activity relationship of flavonoids the study by Ribeiro *et al.* can be reviewed [93].

**Figure 3:**
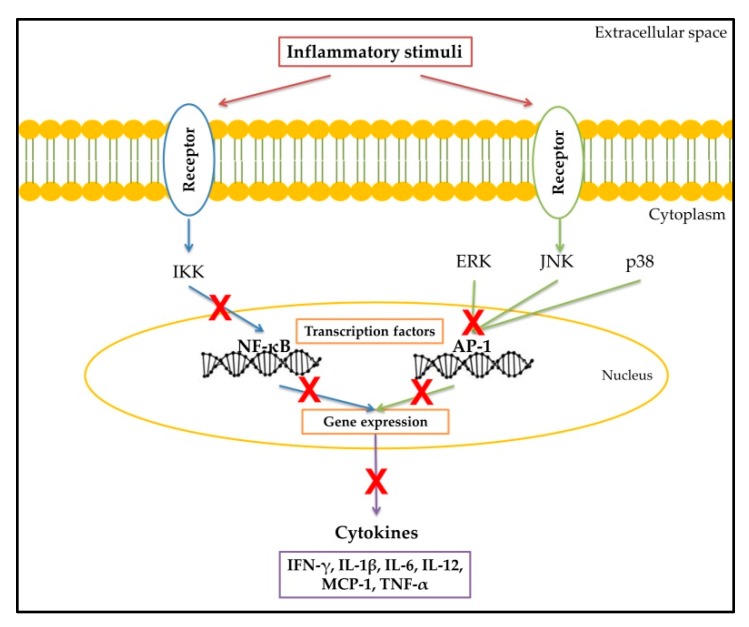
Mechanisms involved in the anti-cytokine effect of flavonoids. The red “X” indicates negative regulation of cytokine synthesis by flavonoids.

This evidence highlights that the anti-inflammatory effect of flavonoids by inhibiting expression and secretion of cytokines, as well as diminishing NF-κB and AP-1 activity, as shown in Figure 3.

## 6. Conclusions

Various inflammatory diseases up-regulate pro-inflammatory cytokines, such as TNF-α and IL-1β, and inflammatory mediators such as NO and prostaglandins, via NF-κB, AP-1 and MAPKs, signal pathways in inflammatory cells. All in all, this manuscript compiled a series of studies that serve as a good basis to support the statement that flavonoids have a promising potential in the development of new drugs to treat inflammation-related diseases. Flavonoids appear to be important modulators of pro-inflammatory cytokines, such as IL-1β, IL-6 and TNF-α. However, the effect of flavonoids on intracellular signaling pathways and on other inflammatory mediators still remains to be investigated, since it would depend on the type of cells, the studied disease and the applied stimulus. Extensive research in this area is therefore required.

## Figures and Tables

**Table 1:** Drugs used to block cytokine activity ^1^.

Therapeutic Agent	Mode of Action	Cytokine Targeted	Reference
Tocilizumab	Anti-IL-6 receptor	IL-6	Oldfield, Dhillon and Plosker [59]
Ustekinumab	Anti-P40	IL-12/IL-23	Papp, *et al.* [63]
Anakinra	IL-1β antagonist	IL-1β	Waugh and Perry [64]
Amgen	Anti-IL-17	TNF-α	Steinman [65]
Etanercept	Soluble receptor	TNF-α	[66,67]
Infliximab	Anti-TNF-α	TNF-α	[67,68]
Dacliqumab	Anti-IL-2 receptor	IL-2	Martin [69]

^1^ Table adapted from Leung, Liu, Fang, Chen, Guo and Zhang [60]. IL: interleukin; TNF: tumor necrosis factor.

**Table 2:** Role of flavonoids as cytokine modulators.

Flavonoid	Effect	Molecular Mechanism Involved	Reference
Apigenin	Reduction of NO and prostaglandin E_2_ (PGE_2_) production. Inhibition of IL-6, IL-1β, IL-12 and TNF-α secretion	Inhibition in the *iNOS*, *COX-2*, *IL-6*, *IL-1β* and *TNF-α* gene expression. Amelioration of p38-MAPK, JNK and ERK phosphorylation	[79,80,87,88,89,96]
Fisetin	Decreased TNF-α, IL-1β, IL-6 and IL-8 expression and production	Inhibited p38, JNK and ERK phosphorylation. Inhibited nuclear translocation of NF-κB	[97]
Luteolin	Reduction of NO, IL-6, MCP-1, TNF-α, IL-1β and IFN-γ production. Stimulation of IL-10 secretion	Reduction of *iNOS* and *COX-2* expression. Inhibition of the JNK and p38 activation. Diminished NF-κB and AP-1 activation	[83,87,89,90,91,93,108]
Naringenin	Diminished NO, MCP-1, IL-6 and TNF-α secretion	Inhibited *iNOS*, *COX-2* and ERK expression	[87,92]
Quercetin	Inhibition of NO, TNF-α, IL-1β, IL-6 and interferon (IFN)-γ production. Increased IL-10 secretion	Suppression in the *COX-2*, *TNF-α*, *IL-1β*, *IL-6* and NF-κB expression. Inhibition of the NF-κB and AP-1 activity	[81,82,87,89,93,94,95,107]

## Abbreviations

AP-1	Activating protein-1
bDMARD	Biological disease-modifying anti-rheumatic drugs
COX	Cyclooxygenase
ERK	Extracellular signal-regulated kinases
IL	Interleukin
iNOS	Nitric oxide synthase inducible
JNK	c-Jun N-terminal kinases
LPS	Lipopolysaccharide
MAPKs	Mitogen-activated protein kinases
MCP-1	Monocyte chemoattractant protein-1
NF-κB	Nuclear factor kappa-light-chain-enhancer of activated B cells
NO	Nitric Oxide
NSAIDs	Nonsteroidal anti-inflammatory drugs
ROS	Reactive oxygen species
TLR	Toll-like receptor
TNF	Tumor necrosis factor
